# Human Epidural AD–MSC Exosomes Improve Function Recovery after Spinal Cord Injury in Rats

**DOI:** 10.3390/biomedicines10030678

**Published:** 2022-03-15

**Authors:** Soo-Eun Sung, Min-Soo Seo, Young-In Kim, Kyung-Ku Kang, Joo-Hee Choi, Sijoon Lee, Minkyoung Sung, Sang-Gu Yim, Ju-Hyeon Lim, Hyun-Gyu Seok, Seung-Yun Yang, Gun-Woo Lee

**Affiliations:** 1 Preclinical Research Center, Daegu-Gyeongbuk Medical Innovation Foundation, Daegu 41061, Korea; sesung@kmedihub.re.kr (S.-E.S.); msseo@kmedihub.re.kr (M.-S.S.); kangkk@kmedihub.re.kr (K.-K.K.); cjh522@kmedihub.re.kr (J.-H.C.); sjlee1013@kmedihub.re.kr (S.L.); tjdalsrud27@kmedihub.re.kr (M.S.); 2 Department of Biomaterials Science (BK21 Four Program), Pusan National University, Miryang 50463, Korea; ysg@pusan.ac.kr; 3 Cellexobio, Co., Ltd., Daegu 42415, Korea; kimyoungin@cellexobio.com; 4 Department of Orthopedic Surgery, Yeungnam University Medical Center, Yeungnam University College of Medicine, 170, Hyochung-ro, Namgu, Daegu 42415, Korea; global_jh@kolmar.co.kr (J.-H.L.); rkaldhkthfl4@yu.ac.kr (H.-G.S.); 5 Company Culture Team, Kolmar Korea Holdings 61, Heolleung-ro 8 gill, Seocho-gu, Seoul 06800, Korea

**Keywords:** spinal cord injury, mesenchymal stem cells, exosomes, extracellular vesicles

## Abstract

Spinal cord injury (SCI) interferes with the normal function of the autonomic nervous system by blocking circuits between the sensory and motor nerves. Although many studies focus on functional recovery after neurological injury, effective neuroregeneration is still being explored. Recently, extracellular vesicles such as exosomes have emerged as cell-free therapeutic agents owing to their ability of cell-to-cell communication. In particular, exosomes released from mesenchymal stem cells (MSCs) have the potential for tissue regeneration and exhibit therapeutic effectiveness in neurological disorders. In this study, we isolated exosomes from human epidural adipose tissue-derived MSCs (hEpi AD–MSCs) using the tangential flow filtration method. The isolated exosomes were analyzed for size, concentration, shape, and major surface markers using nanoparticle tracking analysis, transmission electron microscopy, and flow cytometry. To evaluate their effect on SCI recovery, hEpi AD–MSC exosomes were injected intravenously in SCI-induced rats. hEpi AD–MSC exosomes improved the locomotor function of SCI-induced rats. The results of histopathological and cytokine assays showed that hEpi AD–MSC exosomes regulated inflammatory response. Genetic profiling of the rat spinal cord tissues revealed changes in the expression of inflammation-related genes after exosome administration. Collectively, hEpi AD–MSC exosomes are effective in restoring spinal functions by reducing the inflammatory response.

## 1. Introduction

Spinal cord injury (SCI) represents the loss of sensory, motor, autonomous sympathetic nerves, neuropathic pain, and bowel/bladder dysfunction and is caused by traffic accidents, violence, falls, and sports activities [1,2]. According to a recent study, the incidence of SCI was 0.93 million, with an average of 91,556 new cases of SCI in the United States [3,4]. Loss of function due to SCI depends on the exact anatomical location of the injury and the extent of the damage. SCI is a disease for which there is currently no effective treatment. Therefore, the answer can be found through cell-based therapy or scaffold-based therapeutic strategies and these combinations [5]. This approach allowed researchers to demonstrate that inflammation was lower, spinal cord cavity size was smaller, and axon growth was higher in animal studies [6]. SCI studies have been performed in various animal species, including rats, mice, rabbits, and dogs. The processes occurring in the damaged spinal cord can be divided into four stages: acute, subacute, intermediate, and chronic stages [5]. Rats are most commonly used as spinal cord injury models [7]. In addition, contusion, transection, and compression models are used to damage the rat’s spinal cord. The compression method damages the spinal cord using a device that applies constant pressure such as clip, calibrated forceps, balloon compression, and spinal cord strapping [7,8,9,10]. This is a suitable method for studying secondary injury mechanisms and is recommended for translational research and cell transplantation [7,8,11,12]. Compression injuries are commonly used in SCI rat models because they occur in a manner similar to how SCI occurs in humans.

Exosomes are extracellular vesicles having a bilayer lipid membrane structure, with a diameter of approximately 50–200 nm. Extracellular vesicles are derived from cells and biological fluids, and in particular, exosomes contain mRNA, miRNA, DNA, lipids, proteins, and metabolites, which are secreted through multivesicular bodies and have cell-specific characteristics [13,14,15]. Exosomes are critical mediators of cell-to-cell communication and deliver genetic material derived from the cells of origin. Stem-cell-derived exosomes have become new therapeutic agents, as they have healing and repairing capabilities [16,17,18,19]. As exosomes are safe as cell-free therapeutic agents, clinical trials using exosomes in diseases such as diabetes, chronic liver disease, and ischemic stroke have been reported [20]. Genetic materials in exosomes can have application in effective clinical liquid biopsy by diagnosing cancer or other diseases and predicting prognosis [21]. Exosomes, as a promising delivery strategy targeting the central nervous system (CNS), can cross the blood–brain barrier, a natural barrier [22].

In this study, mesenchymal stem cells (MSCs) were isolated from epidural fat through posterior decompression surgery of the lumbar spine. Exosomes were isolated from human epidural adipose tissue-derived mesenchymal stem cells (hEpi AD–MSCs) using the tangential flow filtration method, and the exosomes were analyzed using transmission electron microscopy (TEM), flow cytometry, and nanoparticle tracking analysis (NTA). The SCI model was established in Sprague Dawley (SD) rats using the compression method, and the exosomes were administered intravenously. Recovery from SCI was observed using the Basso, Beattie, and Bresnahan (BBB) locomotor scale method for four weeks after administration. Subsequently, histological analysis was conducted, and inflammation-related markers such as cytokines were analyzed in the serum and spinal cord tissues. mRNA sequencing was conducted to confirm the changes caused by hEpi AD–MSC exosomes. Our findings suggest that hEpi AD–MSC exosomes alleviated inflammatory responses and are effective therapeutic agents for SCI and diseases/conditions requiring tissue regeneration.

## 2. Materials and Methods

### 2.1. SCI Animal Model

All animal experimental procedures were approved by the Institutional Animal Care and Use Committee of the NDIC Co., Ltd. (IACUC; Approval No. P201103; approved date: 10 February 2020; Hwaseong, Gyeonggi-do, Korea), and all protocols were in accordance with the approved guidelines. In this study, 7-week-old female SD rats, weighing approximately 220 g, were used (Orientbio, Seongnam-si, Gyeonggi-do, Korea). Female rats are preferred in the SCI model because of their ease of bladder emptying and the low risk of urinary tract infections [23,24,25,26]. The animals were acclimatized for one week in an animal facility under controlled temperature and humidity. In total, 24 SD rats were randomly divided into 4 groups (*n* = 6 per group). In the negative control group, the spinal cord was not damaged. In the vehicle group as a positive control, SCI was performed and 0.2 mL phosphate-buffered saline (PBS) was injected intravenously. In the Low-Exo group, SCI was performed and a low dose of exosomes (1 × 10^9^ particles in 0.2 mL PBS) was simultaneously injected intravenously, and the same amount was administered again after 3 days. The High-Exo group was subjected to SCI and intravenous injection of exosomes at a high dose (5 × 10^9^ particles in 0.2 mL phosphate-buffered saline) at the same time intervals. The SCI model was established as described in a previous study [27]. Briefly, the rats were anesthetized using isoflurane (Foran, JW Pharmaceutical, Seoul, Korea). The spinal cord was exposed vertebral column T8-T10, and the T9 spine segment was carefully removed. A 50 g clip–compression injury was performed at the T9. Without interruption of the dura mater or damage to adjacent dorsal and ventral roots, the clip was closed around the cord for 20 s. Behavior tests were conducted using the BBB locomotor scale method before injury (score of 21 points) and on days 3, 7, 14, 21, and 28 after injury. Rats from each group were sacrificed on day 28. Then, spinal cord tissue and serum were collected. For analysis, three spinal cord tissues from each group were fixed in 10% formaldehyde, and the other three spinal cord tissues were frozen for RNA isolation.

### 2.2. Cell Culture and Characterization

This study was approved by the Institutional Review Board of Yeungnam University Medical Center in Daegu, Korea (IRB No. 2017-07-032), and informed consent was obtained from all the patients. hEpi AD–MSCs were isolated from human epidural fat as previously described [28,29]. The cells were cultured in Dulbecco’s modified Eagle’s medium supplemented with 1.0 g/L glucose (DMEM; Gibco, Carlsbad, CA, USA) containing 10% exosome-free fetal bovine serum (FBS; Gibco, Carlsbad, CA, USA) and 1% penicillin–streptomycin (P/S; Gibco, Carlsbad, CA, USA) and incubated at 37 °C with 5% CO_2_. hEpi AD–MSCs at passage 5 to passage 10 were used for further experiments. The expression of stem cell markers on these cells was determined by flow cytometry (FACS; Galios, Beckman Coulter, Brea, CA, USA) using the CD73-PE (BioLegend, San Diego, CA, USA, 344004), CD90-PE (BioLegend, 555596), CD105-PE (Bio-Rad, Hercules, CA, USA, MCA1557), CD14-PE (Bio-Rad, MCA1568), CD34-FITC (BioLegend, 343504), and CD45-FITC (BioLegend, 555482) antibodies.

### 2.3. Isolation and Identification of Exosomes

Exosomes were isolated from the culture medium containing hEpi AD–MSCs. After the cells reached 80% confluence, the medium was collected every 2 d, until 180 mL medium was obtained, which was centrifuged at 300× *g* for 10 min to discard cellular debris. The supernatant was filtered using the tangential flow filtration system (TFF system; Pall Corporation, Port Washington, NY, USA). The feed flow rate was set at 120 rpm. The cell culture medium was concentrated to 3 mL (60 times) using the TFF system. Expression of exosome surface markers was determined by bead-based flow cytometry (FACS; Galios, Beckman Coulter, Brea, CA, USA) using antibodies against CD63 (BioLegend, 353003) and CD81 (BioLegend, 349,505) as previously described [30]. To analyze exosome surface-specific markers, exosomes were mixed with 4% aldehyde/sulfate latex beads (Thermo Fisher Scientific, Rockford, IL, USA). Transmission electron microscopy (TEM; HT7700, Hitachi, Japan) was used to observe morphological characteristics of the exosomes. Exosomes were adsorbed on a formvar carbon-coated copper grid (Ted Pella Inc., Redding, CA, USA), fixed with 2% paraformaldehyde for 10 min, and then dried. Particle size distribution and concentration were determined by NTA (Nanosight NS300, Malvern Panalytical, Worcestershire, UK) following the manufacturer’s instructions.

### 2.4. Histopathological Analysis

The spinal cord tissues were collected from SCI-induced rats for histological analysis on day 28 (*n* = 3 per group). The tissues were fixed in 10% neutral-buffered formalin, embedded in paraffin, and sectioned (thickness: 4 μm). Hematoxylin and eosin staining of the sections was conducted using Dako CoverStainer (Agilent, Santa Clara, CA, USA). The following antibodies were used for immunohistochemistry analysis: anti-ionized calcium-binding adapter molecule 1 (Iba-1) (Abcam, Cambridge, UK) and anti-glial fibrillary acidic protein (GFAP) (Abcam) to determine neuroinflammation. All stained slides were scanned using a Pannoramic SCAN II (3DHISTECH Kft., Budapest, Hungary). Photomicrographs were captured using CaseViewer software 2.5 (3DHISTECH Kft.). The signal was quantified using the ImageJ software (NIH, Bethesda, MD, USA).

### 2.5. Quantitative Polymerase Chain Reaction (qPCR)

Total mRNA was isolated from the spinal cord tissues using RNeasy Mini Kit (Qiagen, Hilden, Germany). The spinal cord tissue was about 10 mm in size and is near the T9 site (*n* = 3 per group). cDNA was reverse-transcribed using Transcriptor First Strand cDNA Synthesis Kit (Roche, Basel, Switzerland) according to the manufacturer’s instructions. Then, qPCR was carried out using LightCycler 480 SYBR Green I Master (Roche) and analyzed on LightCycler 480 system (Roche). The primer sequences of genes encoding glyceraldehyde-3-phosphate dehydrogenase (*GAPDH*), brain-derived neurotrophic factor (*BDNF*), and vascular endothelial growth factor (*VEGF*) are listed in Table 1. The relative expression levels of *BDNF* and *VEGF* were calculated according to the 2^−ΔΔCt^ method.

### 2.6. Cytokine Assay

Rat serum was collected at 28 days after the spinal cord injury and stored at −20 °C for the analysis of cytokines (*n* = 6 per group). Cytokines were detected using Quantibody Rat Cytokine Array (Raybiotech, Norcross, GA, USA) according to the manufacturer’s instructions. The signals were detected using a laser scanner (Innoscan; Innopsys Inc., Carbonne, France) at Cy3 wavelength. Data were analyzed using Mapix software, version 8.2.7 (Innopsys Inc.).

### 2.7. mRNA Sequencing

The isolated RNAs were prepared by pooling for each group for sequencing (*n* = 3 per group). Quantification of the isolated RNA was performed using ND-2000 spectrophotometer (Thermo Inc., Wilmington, DE, USA), and RNA quality was assessed using Agilent 2100 bioanalyzer in combination with RNA 6000 nanochip (Agilent Technologies, Amstelveen, The Netherlands). For control and test RNAs, the library was constructed using a QuantSeq 3′ mRNA-Seq Library Prep Kit (Lexogen Inc., Vienna, Austria) according to the manufacturer’s instructions. Briefly, total RNA (500 ng) was extracted, an oligo-dT primer containing an illumine-compatible sequence at its 5′ end was hybridized to the RNA, and reverse transcription was performed. After degradation of the RNA template, second-strand synthesis was initiated by random primers containing an Illumina-compatible linker sequence at the 5′ end. High-throughput sequencing was conducted using NextSeq500 (Illumina Inc., San Diego, CA, USA). Data analysis was performed using the Bowtie 2 software (Langmead and Salzberg, 2012). Differentially expressed genes were determined based on counts from unique and multiple alignments using coverage in bedtools (Quinlan AR2010, Salt Lake City, UT, USA). The read count data were processed based on the TMM + CPM normalization method using edgeR in the Bioconductor R package (R Development Core Team2020, Vienna, Austria). For gene ontology (GO) analysis, gene classification was based on searches conducted by DAVID Functional Annotation Bioinformatics Microarray Analysis version 6.8 (https://david.abcc.ncifcrf.gov/, accessed on 3 November 2021).

### 2.8. Statistical Analysis

Data are presented as the mean ± standard deviation (SD). Statistically significant differences were analyzed using one-way ANOVA, followed by Tukey’s multiple comparison test using the GraphPad Prism software version 9.3.0 (GraphPad Software, San Diego, CA, USA). Statistical significance was set at *p* < 0.05.

## 3. Results

### 3.1. Isolation and Characterization of hEpi AD–MSC s

**Figure 1:**
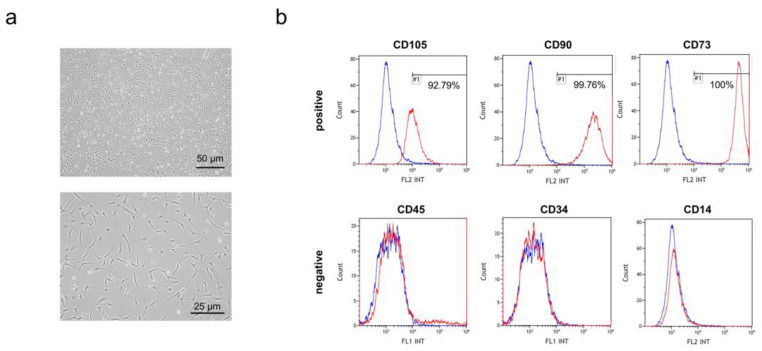
Characterization of human epidural adipose tissue-derived mesenchymal stem cells (hEpi AD–MSCs): (**a**) representative image of passage 5 of human epidural AD–MSCs; (**b**) expression of positive markers (CD105, CD90, and CD73) of human AD–MSCs analyzed by flow cytometry (**upper**). Expression of negative markers (CD45, CD34, and CD14) of human AD–MSCs analyzed by flow cytometry (**below**). The horizontal axis represents the fluorescence intensity (FL1 = FITC, FL2 = PE), and the vertical axis indicates the cell count.

hEpi AD–MSCs were isolated from human epidural fat tissue during posterior decompression surgery of the lumbar spine [29]. According to the criteria for identifying MSCs set by the International Society for Cellular Therapy (ISCT), MSCs adhere to plastic and express specific cell surface markers. The isolated hEpi AD–MSCs were cultured in plastic cell culture dishes and adhered to the bottom of a spindle-shaped morphology (Figure 1a). In addition, hEpi AD–MSCs expressed surface markers CD105, CD73, and CD90, but they did not express CD45, CD34, and CD14 (Figure 1b). hEpi AD–MSCs were cultured until passage 5 to passage 10 to isolate their exosomes from the culture medium.

### 3.2. Isolation and Characterization of Human Epidural AD–MSC Exosomes

**Figure 2:**
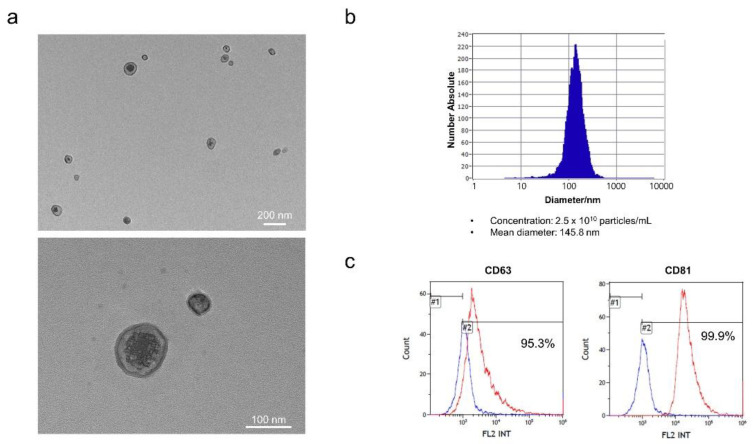
Isolated exosomes from hEpi AD–MSCs: (**a**) representative TEM images of hEpi AD–MSC exosomes; (**b**) the isolated exosomes were analyzed for particle number and size by nanoparticle tracking analysis (NTA); (**c**) the surface positive markers CD63 and CD81 (tetraspanins) expression in exosomes was analyzed by flow cytometry.

The exosomes were isolated from the hEpi AD–MSC culture medium. To isolate exosomes from the culture medium, filtration was performed using TFF. After filtration to a specific size under constant pressure and fluid velocity, the extracellular vesicles remained in the concentrated medium. To eliminate the microparticles, the concentrated medium was centrifuged. Approximately 3 mL of hEpi AD–MSC exosomes were isolated from the supernatant. The isolated exosomes were characterized. Exosomes are small membrane-bound lipid vesicles with diameters ranging from 50 to 200 nm. Microscopy-based methods such as scanning electron microscopy (SEM), transmission electron microscopy (TEM), and cryoelectron microscopy (Cryo-EM) can reveal exosomes at high resolution [31]. The isolated hEpi AD–MSC exosomes were visualized by TEM (Figure 2a). NTA, a particle tracking method, was used to determine the size distribution and concentration of exosomes. The isolated hEpi AD–MSC exosomes had an average diameter of 145.8 nm and a concentration of 2.5 × 10^10^ particles/mL (Figure 2b). Exosomes contain proteins derived from cellular membranes and intracellular organelles. Exosome membrane proteins such as tetraspanins CD63 and CD81 were analyzed using bead-based flow cytometry (Figure 2c). Collectively, nanoparticles with an average size of 145.8 nm cup-shaped were positive for CD63 and CD81. Therefore, this study showed that human epidural AD–MSC exosomes have typical characteristics of exosomes.

### 3.3. hEpi AD–MSC Exosomes Improved SCI in a Rat Model

**Figure 3:**
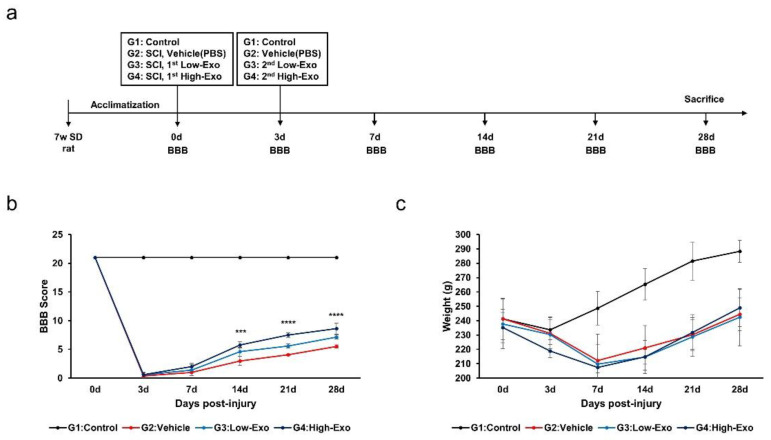
In vivo evaluation of hEpi AD–MSC exosomes for SCI treatment: (**a**) schematic representation of SCI rat model with exosome injection; (**b**) hindlimb locomotor function was measured using the BBB scoring method for 28 d. Score ranged from 0 to 21 depending on the function of hindlimb (*** *p* ≤ 0.001, **** *p* ≤ 0.0001 between the Vehicle and High-Exo group); (**c**) mean body weight in non-treated and SCI-induced rat model (*n* = 6, per group).

To investigate whether hEpi AD–MSC exosomes ameliorate SCI symptoms, we evaluated the effects of hEpi AD–MSC exosomes in a rat model. We used SCI-induced SD rats that were injured at the T9 site using a compression clip and injected hEpi AD–MSC exosomes at low and high concentrations via the tail vein. Three days later, the same amount of hEpi AD–MSC exosomes were intravenously injected again. The control group underwent only laminectomy, and the vehicle group was not administered exosomes; BBB locomotor evaluation was performed on days 0, 3, 7, 14, 21, and 28 after SCI (Figure 3a). The BBB locomotor assessment is scored on a scale of 0–21 representing sequential stages during recovery after SCI such as movement of joints, hindlimb and forelimb coordination, trunk stability, and paw and tail position [32,33]. We evaluated the effects of hEpi AD–MSC exosomes using the BBB scoring (Figure 3b). All rats scored 21 before SCI; however, after SCI, the functional behavior worsened in the Vehicle (0.39 points), Low-Exo (0.56 points), and High-Exo (0.61 points) groups. Seven days after administration of hEpi AD–MSC exosomes, the functional behavior improved in the Vehicle (1.0), Low-Exo (1.39), and High-Exo (2.0) groups, and it was significantly recovered in a dose-dependent manner from day 14 (Vehicle (3.0), Low-Exo (4.6), and High-Exo (5.78) on day 14; Vehicle (4.07), Low-Exo (5.60), and High-Exo (7.50) on day 21; Vehicle (5.53), Low-Exo (7.13), and High-Exo (8.61) on day 28). A score of 8 or more indicated extensive hindlimb movement in the BBB scoring. Body weight did not differ significantly among all SCI groups (Figure 3c). However, the average body weight increased on day 28 in the High-Exo group. All *p*-value values are listed in Table 2.

### 3.4. Histopathological Evaluation

**Figure 4:**
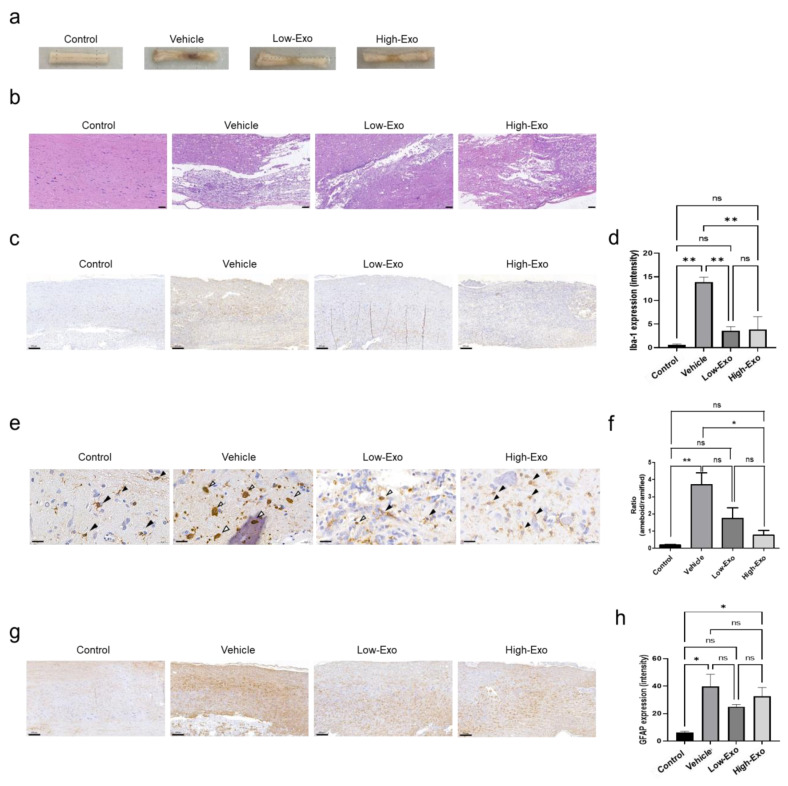
Histopathology and immunohistochemistry analysis of Iba-1 and glial fibrillary acidic protein (GFAP) in the rat spinal cord: (**a**) representative images of spinal cord gross findings after sacrifice. The spinal cord tissues were damaged by compression stimulation; (**b**) the spinal cord tissues were sectioned longitudinally and stained with hematoxylin and eosin. (Scale bar = 100 μm); (**c**) immunodetection of Iba-1 in the non-treated and SCI-induced rats (scale bar = 200 μm); (**d**) quantification of Iba-1 expression by DAB staining; (**e**) representative images of Iba-1 microglia states (scale bar = 20 μm, black arrow: ramified, white arrow: ameboid); (**f**) quantification of Iba-1 morphology ratio (ameboid and ramified microglia); (**g**) immunodetection of GFAP in the non-treated and spinal cord injured rats. (Scale bar = 200 μm); (**h**) quantification of GFAP expressing intensity by DAB staining. (* *p* ≤ 0.05, ** *p* ≤ 0.001, ns = not significant).

The gross findings and histological analysis of the spinal cord showed damaged tissue following compression stimulation (Figure 4a,b). Severe SCI was observed in the vehicle group. In contrast, the exosome-treated groups showed tissue recovery. To evaluate neuroinflammation, tissue immunostaining was performed for immunohistochemical Iba-1 and GFAP in the spinal cord tissues. Iba-1 is a gene specifically expressed in microglia and macrophages, also known as allograft inflammatory factors [34]. Compared with that in the control group, Iba-1 expression was increased in the SCI group. However, Iba-1 expression was significantly decreased in the Low-Exo and High-Exo groups (Figure 4c,d). The morphology of microglia in the spinal cord changes after damage. The ramified form was observed in the restoring state without damage, but ameboid shapes, which are activated states, were observed in the damaged groups. We measured the ratio of the ameboid form to the ramified form. As a result, it was confirmed that the ameboid shape decreased when exosomes were treated at low and high concentrations (Figure 4e,f). GFAP, a hallmark of astrocytes, expression was increased in the vehicle group, whereas it was decreased in the Low-Exo and High-Exo groups, although the difference was not significant (Figure 4g,h). Although Iba-1 and GFAP did not change as much as the negative control group in the exosome-treated group, it was confirmed that they decreased compared to the vehicle group. Taken together, it was possible to confirm the changes in Iba-1 and GFAP in the spinal cord tissue when exosomes were applied at low and high concentrations and to confirm that exosomes were helpful in reducing neuroinflammation.

### 3.5. hEpi AD–MSC Exosomes Increased the Expression of Neurotrophin Factor

**Figure 5:**
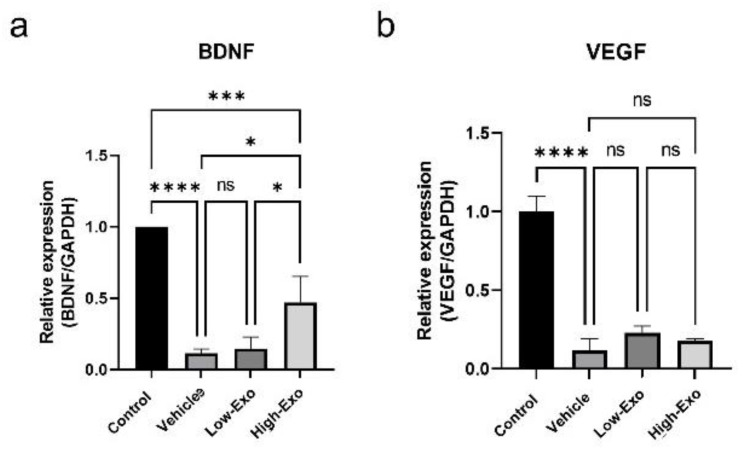
Gene expression levels in rats of the control and SCI groups: (**a**,**b**) expression patterns of BDNF and VEGF measured by qPCR (* *p* ≤ 0.05, *** *p* ≤ 0.001, **** *p* ≤ 0.0001, ns = not significant).

Neuroregeneration substances are required to regenerate damaged nerves. Neurotrophins help to create a regenerative environment for nerves. BDNF exerts neuroprotective and growth-promoting effects on various injured neurons [35]. A comparison of BDNF expression levels in the spinal cord tissues revealed that BDNF level was significantly reduced in the SCI-induced group, compared with that in the control group. However, it was significantly increased in the High-Exo group (Figure 5a). VEGF serves as a revascularization agent for angiogenesis and SCI. VEGF has been considered a potent neurotrophic factor for the survival of spinal neurons [36]. VEGF expression level in the exosome-treated groups was higher than that in the vehicle group, but the difference was not significant (Figure 5b).

### 3.6. hEpi AD–MSC Exosomes Reduced the Expression of Inflammatory Factors

**Figure 6:**
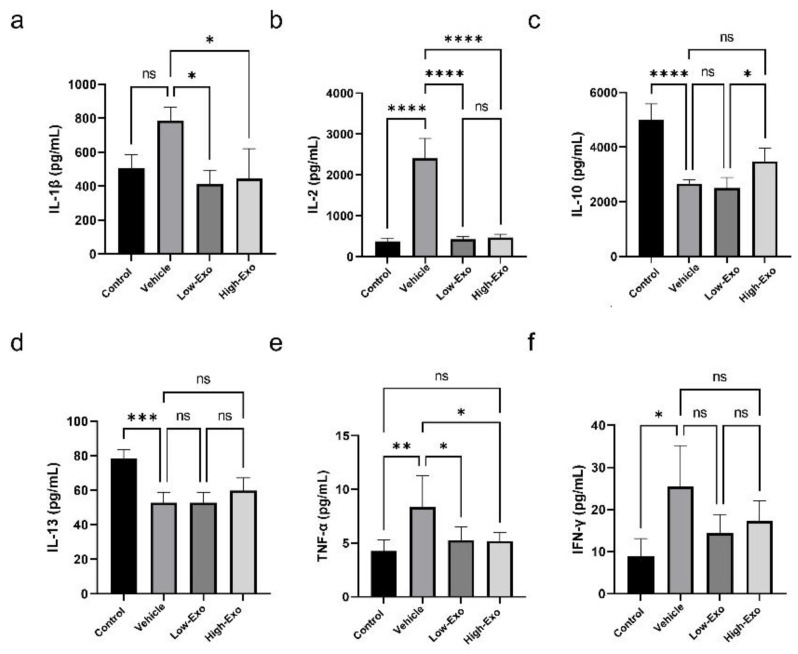
Cytokine levels in rats of the control and SCI groups: (**a**–**f**) levels of cytokines IL-1β, IL-2, IL-10, IL-13, TNF-α, and IFN-γ were analyzed in the serum of the control and SCI-induced rats (* *p* ≤ 0.05, ** *p* ≤ 0.01, *** *p* ≤ 0.001, **** *p* ≤ 0.0001, ns = not significant).

SCI causes severe damage to the central nervous system, followed by an immediate secondary inflammatory response in the spinal cord tissues. Our findings showed that hEpi AD–MSC exosomes regulated the expression of pro-inflammatory and anti-inflammatory cytokines in SCI-induced rats (Figure 6a–f). Pro-inflammatory cytokines interleukin (IL)-1β, IL-2, and tumor necrosis factor (TNF)-α increased significantly when SCI occurred, and significantly decreased in exosome-treated groups. Although there was no significant difference, compared with the vehicle group, the mean value of interferon (IFN)- γ decreased after exosome treatment. In addition, when SCI occurred, anti-inflammatory cytokines IL-10 and IL-13 were significantly decreased, compared with the control group. Anti-inflammatory cytokines increased when treating high concentration exosomes, but it was not significant, compared with the vehicle group. These results suggest that hEpi AD–MSC exosomes reduce the inflammatory response of spinal cord injury through the regulation of various cytokines. 

### 3.7. Comparison of Gene Expression in the Spinal Cord Tissues

**Figure 7:**
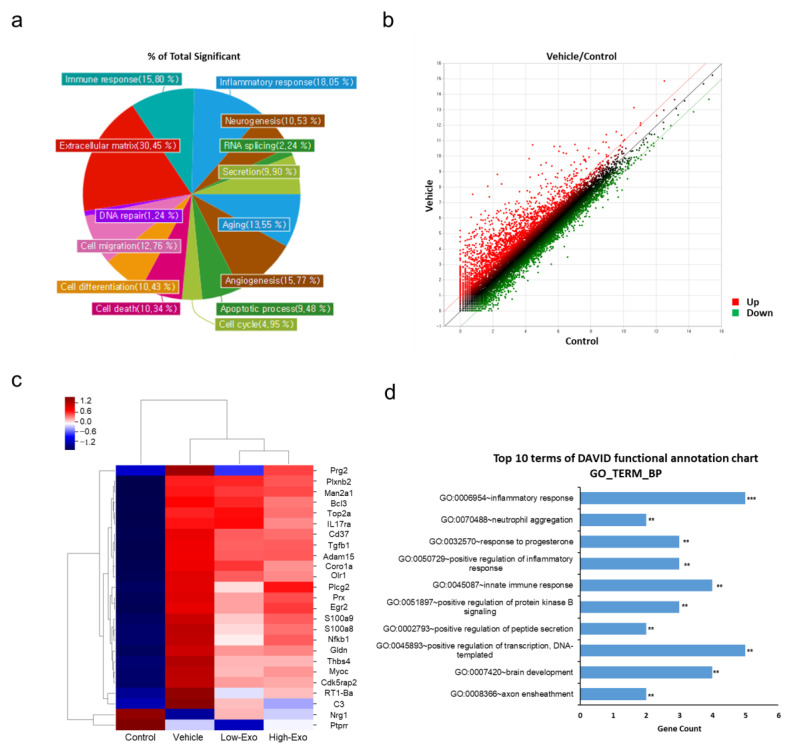
Comparison of gene expression level by mRNA sequencing: (**a**) gene category chart of the control and vehicle groups. The ratio of significant genes according to each gene category; (**b**) scatter plot of normalized data (log2N) for each gene. X-axis represents control (log2N), and y-axis represents vehicle value (log2N), upregulated genes (red) and downregulated genes (green); (**c**) clustering heatmap for genes with significantly altered expression; (**d**) top 10 Gene Ontology (GO) biological process (BP) terms of genes with significantly altered expression in the exosome-treated groups, compared with that in the vehicle group (** *p* < 0.01, *** *p* < 0.001).

mRNA sequencing was performed to observe changes in gene expression in the SCI-induced and hEpi AD–MSC exosome-treated groups. In the control and SCI-induced groups, 1064 genes were confirmed to have altered expression. The majority of changes occurred in the genes related to the extracellular matrix, inflammatory response, and immune response (Figure 7a). According to the scatter plot data, the expression of more genes was upregulated in the vehicle group than in the control group. There were 793 upregulated genes (red spots) and 271 downregulated genes (green spots) (Figure 7b). The differences in the expression of 25 genes (*Prg2*, *Plxnb2*, *Man2a1*, *Bcl3*, *Top2a*, *IL17ra*, *Cd37*, *Tgfb1*, *Adam15*, *Coro1a*, *Olr1*, *Plcg2*, *Prx*, *Egr2*, *S100a8*, *S100a9*, *Nfkb1*, *Gldn*, *Thbs4*, *Myoc*, *Cdk5rap2*, *RT1-Ba*, *C3*, *Nrg1*, and *Ptprr*) were visualized through clustering (Figure 7c). These genes belong to the categories related to immune response, inflammatory response, and neurogenesis. In addition, these 25 genes with significantly altered expression in the exosome-treated group, compared with that in the vehicle group, were analyzed to predict functional annotations (Figure 7d). Our findings show that hEpi AD–MSC exosomes played a role in reducing SCI-induced inflammatory responses by targeting immune response and neurogenesis-related genes in the SCI rat model.

## 4. Discussion

Extracellular vesicles are classified into three subtypes based on their origin: exosomes, microparticles, and apoptotic bodies. They differ in origin, size, and biological properties; exosomes smaller than 200 nm can be isolated using ultracentrifugation, filtration, polyethylene glycol (PEG), size-exclusion chromatography, and microfluidic immunoaffinity [37]. However, exosome isolation technology requires innovative strategies and devices that have the advantages of being rapid and cost-effective, with efficient isolation, high concentration, and high purity [38]. Although ultracentrifugation is the most commonly used method to concentrate exosomes, TFF is a more efficient, scalable, and gentle method that does not damage exosomes. It is also suitable for the large-scale production of high-quality exosomes compliant with good manufacturing practices (GMPs) [39,40,41]. Considering the advantages of TFF, we used the TFF method to isolate a sufficient quantity of exosomes from a large amount of cell culture medium for characterization and in vivo application. In addition, the exosomes isolated using the TFF method did not undergo functional or morphological modifications.

We used MSCs for the isolation of exosomes because they are more accessible than embryonic stem cells, without any concern about tumor development and ethical issues, and have rapid proliferation and multi-lineage differentiation capabilities [42,43,44]. Therefore, MSCs have been used in research to reduce inflammation and damage in spinal cord injury. Exosomes derived from MSCs are used as drug delivery systems because they are easier to preserve than MSCs. [45]. Epidural adipose tissues are routinely eliminated during posterior decompression surgery of the lumbar spine; however, epidural adipose removal causes certain problems, such as post-laminectomy syndrome [29,46]. We hypothesized that epidural fat may play a key role in the neural structure and nearby conditions. We demonstrated the anti-inflammatory effects of hEpi AD–MSC-derived exosomes compared to dermal fibroblasts-derived exosomes. As a result, it was confirmed that the hEpi AD–MSC-derived exosomes inhibit the release of pro-inflammatory cytokines such as TNF-α and IL-6 in LPS-induced inflammation in THP-1 macrophages [28]. Due to these advantages of exosomes, recent studies have been reported using exosomes derived from MSCs to treat SCI [44]. A recent study suggested that bone MSC-derived extracellular vesicles reduce brain cell death and improve motor function, in addition to improving the integrity of the blood–spinal cord barrier by reducing the movement of pericytes through downregulation of the NF-κB p65 signaling [27]. Exosomes help in the recovery of spinal cord damage due to their anti-inflammatory and anti-apoptotic effects exhibited through mechanisms of A1 astrocyte inhibition, axonal regenerative promotion, and macrophage polarization [47]. The anti-inflammatory effect of exosomes derived from MSCs is related to the relatively reduced levels of pro-inflammatory cytokines such as IL-1β, IL-6, and TNF-α [48,49,50]. The nucleotide-binding domain-like receptor protein (NLRP3) inflammasome plays an important role in the secondary damage caused by SCI. Inflammasomes are multi-protein complexes that trigger the activation of caspase-1, followed by the maturation of pro-inflammatory cytokines [51]. Pharmacological suppression of NLRP3 inflammasome activation regulates neuroinflammation, attenuates mitochondrial dysfunction, and enhances functional recovery after SCI. Exosomes derived from MSCs inhibit the activation of the NLRP3 inflammasome and promote neurological recovery in SCI rats [52,53]. hEpi AD–MSC extracellular vesicles reduced the lesion volume and cell apoptosis after SCI [53]. NLRP3 inflammasome has a crucial role in the secretion and mature of cytokines IL-1β and IL-18 by triggering the activation of caspase-1. Additionally, the NLRP3 inflammasome could be a promising therapeutic target for the CNS diseases such as SCI, traumatic brain injury, and ischemic stroke [51]. The expression pattern of mRNA in the rat SCI model and exosome application has not yet been reported. Therefore, it is expected that the present study can provide new insights into neurological disease research by correlating the therapeutic effect of hEpi AD–MSC exosomes with changes in mRNA expression levels in SCI. Indeed, when the SCI and control groups were compared, it was confirmed by mRNA sequencing that the expression of genes related to the extracellular matrix, inflammatory response, and immune response was changed significantly (Figure 7a). The biological function analysis of 25 genes that were significantly altered after exosome treatment confirmed that exosomes regulated inflammatory response in SCI (Figure 7c,d). Thrombospondin-4 (Thbs4) is one of the five members of the thrombospondin family [54]. Thbs4 supported local vascular inflammation in the atherosclerosis model and was associated with inflammation. Thbs4^−/−^ mice showed decreased expression of pro-inflammatory factors [55]. Bcl3 is mainly localized in the nucleus and regulates NF-κB transcriptional activity. Bcl3 expression was induced by IL-6 in multiple myeloma cells, which increased apoptosis [56]. It was associated with IL-8 and IL-17 in skin T-cell lymphoma cells to regulate survival and inflammatory gene expression [57]. It was confirmed that an increase in Thbs4 and Bcl3 levels in SCI was reversed after treatment with hEpi AD–MSC exosomes (Figure 7c). Moreover, C3 expression has been reported to increase in chronic inflammation [58], and when exosomes were applied to these SCI models, the decrease in C3 expression showed that exosomes were effective against inflammation. These findings are expected to have an effect on exosomes in both the acute stage and the chronic stage of SCI. Biodistribution, through fluorescent dye labeling on exosomes, shows the distribution of administered exosomes in vivo [51,59,60,61]. Since MSCs target damaged tissues, MSCs-derived exosomes also accumulate in the damaged spinal cord [61]. As a result, inflammatory responses are reduced and tissue regeneration. Currently, there is no approved therapy available to restore motor and sensory nerves after SCI, which necessitates the development of new strategies to promote axonal regrowth and restore neuronal function. Therefore, it is necessary to understand the cellular and molecular mechanisms that impair regeneration or neuroplasticity [62]. Systemic application through intravenous injection is limited by the concentration of exosomes that can be used. Ideal drug delivery should enable target-specific delivery of therapeutic agents. Exosomes can be classified into two strategies for drug delivery: cargo loading and exosome modification [45]. Representative methods for encapsulating cargo are incubation, electroporation, and sonication, and cargo types include drugs, nucleic acids, peptides, and nanomaterials. For instance, anti-cancer drugs, doxorubicin and paclitaxel, can be loaded into exosomes and delivered to target tissues [63]. A study demonstrated that BDNF was loaded into macrophage-derived exosomes and applied for Parkinson’s disease therapy [64]. Exosomes are biocompatible and prevent recognition and degradation by the immune system. Although many advantages of exosomes, there is a limit to the loading efficiency of the cargos. Moreover, as a result of in vivo, exosomes are targeting the kidneys, liver, and other organs. Thus, in order to increase delivery function, engineering exosomes by fusion with liposomes. Liposomes have suitable properties for drug delivery due to their lipid bilayer structure. The result of exosome–liposome hybrid nanoparticles suggested that decreased immunogenicity and increased colloidal stability and improved the half-life of exosomes in blood [65]. Additionally, there are several approaches of modifying exosomes to obtain targeting ability: ligand-receptor binding, pH sensitivity, surface charge, and magnetic field [66]. The improved exosome delivery system can be used to treat patients with neurological dysfunction diseases through clinical trials.

## 5. Conclusions

In conclusion, hEpi AD–MSC exosomes have therapeutic potential, as they regenerate damaged tissue and restore motor function by alleviating inflammation in SCI. To the best of our knowledge, this is the first report to compare the differences in gene expression and analyze the functions of critical genes when hEpi AD–MSC exosomes were applied in SCI, a neurological injury.

## Figures and Tables

**Table 1:** Primer sequences list.

	Forward	Reverse	Product Size
BDNF	TGGAAAGGGTGAAACAAAGTG	TAATGTTGTCAAACGGCACAA	183 bp
VEGF	GAGGAAAGGGAAAGGGTCAAA	CACAGTGAACGCTCCAGGATT	69 bp
GAPDH	TACCAGGGCTGCCTTCTCTT	GATCTCGCTCCTGGAAGATG	191 bp

**Table 2:** The *p*-value for BBB scoring and body weight.

	BBB Scores *p*-Value			Body Weight *p*-Value		
	Vehicle vs. Low-Exo	Vehicle vs. High-Exo	Low-Exo vs. High-Exo	Vehicle vs. Low-Exo	Vehicle vs. High-Exo	Low-Exo vs. High-Exo
Day14	0.0169	0.0002	0.0671	0.478	0.379	0.964
Day21	<0.0001	<0.0001	<0.0001	0.845	0.835	0.708
Day28	0.0091	<0.0001	0.0117	0.847	0.560	0.525

## Data Availability

The data that support the findings of this study are available from the corresponding author upon reasonable request.
